# Tissue and plasma putrescine levels in non-survivors of sepsis in a fluid-resuscitated rat model of faecal peritonitis

**DOI:** 10.1186/2197-425X-3-S1-A616

**Published:** 2015-10-01

**Authors:** DT Andreis, W Khaliq, S Neugebauer, M Kiehntopf, M Singer

**Affiliations:** University College London, Bloomsbury Institute of Intensive Care Medicine, London, United Kingdom; Dipartimento di Fisiopatologia Medico-Chirurgica e dei Trapianti, Università degli Studi di Milano, Milan, Italy; Institut für Klinische Chemie und Laboratoriumsdiagnostik, University Hospital Jena, Jena, Germany

## Introduction

The polyamine, putrescine, was first isolated from putrefying meat but is thought to play an important role in cell growth and differentiation ([1]). It also generates succinate via GABA and can thus serve as an energy source to the small intestine ([2]). Elevated plasma putrescine levels have been reported in an endotoxic rodent model ([3]). We have previously characterized a 72 h fluid-resuscitated rat model of faecal peritonitis where prognostication can be accurately made as early as 6 h post-insult ([4]).

## Objectives

Using this long-term sepsis model, to assess differences in liver and plasma levels of putrescine in predicted survivors and non-survivors.

## Methods

Awake, instrumented yet fully mobile male Wistar rats (325 ± 15 g) received an i.p. injection of 4µl/g faecal slurry. Fluid resuscitation (50:50 mix of 5% glucose/Hartmann's; 10 ml/kg/h) was commenced at 2 h. At 6 h, an echo-measured heart rate cut-off of 460 bpm was used to classify animals into predicted survivors or non-survivors. Animals were sacrificed at 6 h, 24 h or 72 h for liver and blood sampling. A group of control animals were treated identically but without injection of faecal slurry. Putrescine levels were measured using mass spectrometry. Results were analysed using two-way ANOVA and post-hoc testing and considered statistically significant when p < 0.05.

## Results

In this model septic animals had a mortality rate of 56% with death occurring between 18-36 h. At 6 h septic animals displayed only mild clinical features of illness. However, even as early as 6 h, significant differences were noted in putrescine levels in liver and plasma from non-surviving septic animals.

## Conclusions

An association was seen between eventual non-survival and elevated putrescine levels in both liver and plasma at both 6 h and 24 h. The significance of this finding warrants further investigation.

**Table 1:** Table 1

Putrescine level (µmol/L)		Control	Septic Survivors	Septic Non-Survivors
6 h	Plasma	1.59 ± 0.20	1.91 ± 0.24	3.51 ± 0.42*
	Liver	0.29 ± 0.06	0.41 ± 0.13	3.58 ± 0.63*
24 h	Plasma	1.39 ± 0.10	1.68 ± 0.17	4.20 ± 0.82*
	Liver	0.44 ± 0.21	0.42 ± 0.16	3.61 ± 1.45*
72 h	Plasma	1.28 ± 0.09	1.20 ± 0.12	-
	Liver	0.47 ± 0.10	0.31 ± 0.09	-
Data shown as median ± SE; * p < 0.05				

## Grant Acknowledgement

ESICM Basic Science Award, Intensive Care Foundation (UK), NIHR
